# Peer Review of “Why We Are Losing the War Against COVID-19 on the Data Front and How to Reverse the Situation”

**DOI:** 10.2196/28722

**Published:** 2021-05-05

**Authors:** Laura Lafon-Hughes

**Affiliations:** 1 Departamento de Genética Instituto de Investigaciones Biológicas Clemente Estable Montevideo Uruguay; 2 Grupo de Biofisicoquímica Departamento de Ciencias Biológicas Centro Universitario Regional Litoral Norte, Universidad de la República Salto Uruguay

**Keywords:** COVID-19, learning health systems

*This is a peer-review report submitted for the paper “Why We Are Losing the War Against COVID-19 on the Data Front and How to Reverse the Situation”.*

## Round 1 Review

### General Comments

This paper [1] addresses a pressing issue: pandemic data collection and processing.

The paper provides a clear diagnosis of why we are losing the war against COVID-19 on the data front (first part of the title), including all types of variables like technical aspects, medical staff overload and lack of time to fill in forms, ethical issues management, and bottlenecks for project presentations and analysis. However, the “how to reverse the situation” section (the second part of the title) is not in-depth and could be readdressed from a more practical, useful point of view. Below are some suggestions.

### Specific Comments

1. It is true that health staff are overloaded. Discuss alternative solutions regarding who could fill in the forms. For example, could the patient or patient’s family complete a form on a cellphone, using an identifier to track the patient but not the person themselves and send the data to the system? In so doing, the patient would be providing consent to use the data for research purposes. Alternatively, should researchers be recruited to work alongside doctors and nurses and complete the forms, which they can analyze at the same time?

2. Please suggest a structure for the data set. What important information should be recruited? What would a universal data sheet look like? Here are some variables I can rapidly think of. Discuss whether these should be included or not and why, and add other ones. The data set could take into account the long-term effects that may occur.

Patient-specific code: ___________________

*To be filled in by the patient or family*

- Country, region, or city; urban/rural
- Age
- Biological sex
- Population ancestry (Africa, Asia, Europe, the Americas)
- Preexistent conditions: allergy, immunodepression, AIDS, frequent respiratory illness, diabetes, hypertension, heart attack, heart surgery, renal problems, liver problems, gastrointestinal problems, psychiatric problems (depression, anxiety, other)
- Care availability, running water supply and soap to wash hands, exposure to extreme temperatures, mask wearing, general living conditions, unemployment, hunger
- Symptoms: a cold, a sore throat, fever, diarrhea, cardiac, neural (loss of taste or smell, dizziness, disorientation)
- Vaccine received? Specify which one and when. Important: People should be instructed to retain their vaccine data, lab, lot, etc, even in clinical trials

*To be completed by medical staff*

- COVID-19–positive personal contacts
- Days before symptom onset
- Open or closed space
- Mask wearing: yes or no
- Diagnosis confirmed by polymerase chain reaction (day after first symptoms)
- Antibody test (day after symptoms)
- Clinic and paraclinic pneumonia
- ACV
- Blood clots
- Renal failure
- Liver failure
- Treatments attempted (to be completed by doctors)
- Respirator (which one, how many days)
- Anti-inflammatory therapy (which one, how much, when)
- Nonspecific antiviral therapy, for example, remdesivir (which one, how much, when)
- Specific anticoronavirus therapy
- Days in hospital
- Survival?
- Relapse (when, how)
- Long-term changes that may or may not be related to COVID-19
- Neurodegeneration
- Liver failure
- Heart failure
- Lack of strength
- No preexistent depression
- No preexistent diabetes

3. Can you suggest existent public database platforms that could be immediately used for data collection?

4. Do you or does somebody you know have software ready or one that can be easily developed to perform the automatic data processing? Maybe Johns Hopkins University could help? (It is just an idea because they have been automatically collecting COVID-19 diagnosis and deaths by country.)
